# Fetal movement in late pregnancy – a content analysis of women’s experiences of how their unborn baby moved less or differently

**DOI:** 10.1186/s12884-016-0922-z

**Published:** 2016-06-01

**Authors:** Anders Linde, Susanne Georgsson, Karin Pettersson, Sofia Holmström, Emma Norberg, Ingela Rådestad

**Affiliations:** Department of Clinical Science, Intervention and Technology, Karolinska Institutet, Stockholm, Sweden; Sophiahemmet University, PB 5605, S-114 86 Stockholm, Sweden

**Keywords:** Pregnancy, Fetal movement, Decreased fetal movements, Content analysis

## Abstract

**Background:**

Pregnant women sometimes worry about their unborn baby’s health, often due to decreased fetal movements. The aim of this study was to examine how women, who consulted health care due to decreased fetal movements, describe how the baby had moved less or differently.

**Methods:**

Women were recruited from all seven delivery wards in Stockholm, Sweden, during 1/1 – 31/12 2014. The women completed a questionnaire after it was verified that the pregnancy was viable. A modified content analysis was used to analyse 876 questionnaires with the women’s responses to, “Try to describe how your baby has moved less or had changes in movement”.

**Results:**

Four categories and six subcategories were identified: “Frequency” (decreased frequency, absence of kicks and movement), “Intensity” (weaker fetal movements, indistinct fetal movements), “Character” (changed pattern of movements, slower movements) and “Duration”. In addition to the responses categorised in accordance with the question, the women also mentioned how they had tried to stimulate the fetus to move and that they had difficulty in distinguishing fetal movements from contractions. Further, they described worry due to incidents related to changed pattern of fetal movements.

**Conclusion:**

Women reported changes in fetal movement concerning frequency, intensity, character and duration. The challenge from a clinical perspective is to inform pregnant women about fetal movements with the goal of minimizing unnecessary consultations whilst at the same time diminishing the length of pre-hospital delay if the fetus is at risk of fetal compromise.

**Trial registration:**

Not applicable.

**Electronic supplementary material:**

The online version of this article (doi:10.1186/s12884-016-0922-z) contains supplementary material, which is available to authorized users.

## Background

It is widely acknowledged that a pattern of regular movements is associated with fetal wellbeing [1]. Fetal movements can be defined as any discrete kick, flutter, swish or roll and are normally first perceived by the mother between 18 and 20 weeks of gestation [2]. The frequency of fetal movements reaches a plateau in gestational week 32 and stays at that level until birth [3]. There is normally a variation in fetal movements with a wide range in the number of movements per hour [4]. The movements are normally absent during sleep and occur regularly throughout the day and night, normally lasting for 20–40 min. The sleep cycles rarely exceed 90 min in the normal and healthy fetus [5]. Although the movement pattern of the individual fetus is unique, it is general knowledge that decreased fetal movement is associated with adverse outcome, including stillbirth [6].

The character of the movements changes when the pregnancy approaches delivery due to limited space in the uterus, but the frequency and intensity will not normally decrease [3]. In an interview study, 40 term pregnant women with an outcome of a healthy baby described fetal movements during the past week. Almost all experienced fetal movements as “strong and powerful”. Half of the women also described the movements as “large” (involving the whole body of the fetus). Another common description was “slow” as in “slow motion” and “stretching” or “turning”. Some of the women stated that they were surprised how powerfully the fetus moved [7].

Several maternal factors may impair the ability to recognize fetal movement [8]. Amniotic fluid volume [9], fetal position [10], having an anterior placenta [10, 11], smoking, being overweight [6] and nulliparity [6, 12] have been reported as such factors. Maternal factors which may enhance the ability to recognize movement are the opportunity to focus on the fetus and the absence of distracting noises [13]. About 50 % of the pregnant women in a study from Norway were sometimes worried about decreased fetal movements [14]. In a review article, it was found that between four and fifteen percent of pregnant women consult health care because of a decrease in fetal movement in the third trimester [1]. The aim of the present study was to examine how women, who consulted health care due to decreased fetal movements after gestational week 28, describe how the baby had moved less or differently.

## Methods

### Settings and participants

**Fig. 1:**
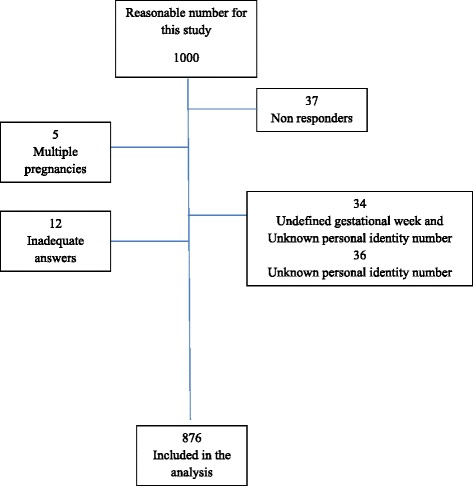
Flow chart

Women were recruited from all seven delivery wards in Stockholm, Sweden from 1st January to 31st December 2014, and were asked to complete a questionnaire. The inclusion criteria were women in gestational week 28 or more who consulted health care due to concerns over decreased fetal movements, with the ability to understand Swedish or English and a normal cardiotocography (CTG). Non responders, inadequate answers, multiple pregnancies, undefined gestational week and unknown personal identity number were exclusion criteria (Fig. 1). In total, 3555 questionnaires were completed during the data collection period. Data collection was in progress while the first 1000 questionnaires were analysed. Twenty-eight women completed two questionnaires and three women filled in three questionnaires; they consulted health care more than once during the data collection period due to concerns over decreased fetal movements. Of the women, 672 (76.7 %) were aged 20–35 years, 582 (66.4 %) had a college or university level of education and 650 (74.2 %) of the women were born in Sweden (Table 1). All women gave birth to a live child.

**Table 1:** Age, level of education and country of birth among the 876 women in the study

Age, level of education and country of birth among the 876 women in the study	n (%)
Age (yr)^a^	
< 20	2 (0.2)
20–35	672 (76.7)
> 35	202 (23.1)
Education^b^	
Elementary school	28 (3.2)
High school	258 (29.4)
College or university	582 (66.4)
Country of birth^c^	
Sweden	650 (74.2)
Europe (excluding Sweden)	86 (9.8)
Asia	90 (10.3)
Africa	19 (2.2)
South America	20 (2.3)
North America	6 (0.7)
Australia/New Zeeland	1 (0.1)

^a^Non responders, 0 (0.0); ^b^Non responders, 8 (0.9): ^c^Non responders, 4 (0.4)

### Data collection

The questionnaire used in the study was developed from a web survey, an interview study [7, 15] and clinical experience. The questionnaire was face-to-face validated with ten women who consulted health care due to reduced fetal movements, not included in the study. The final version of the questionnaire included a total of 22 questions with multiple-choice or open-ended response alternatives (Additional file 1). This study comprises the women’s responses to the request: “Try to describe how your baby has moved less or had changes in movement”. The women were asked to describe their experiences in the space provided but could also, if necessary, continue on the back of the questionnaire.

### Analysis

The women’s descriptions (*n* = 876) of how their unborn baby had moved less or differently were analysed using a modified content analysis [16]. The material consisted of concise descriptions of movements, which were used without editing. The analysis was performed in three steps. Firstly, all the answers were read and re-read three times to gain a sense of content in the data. Codes were then revealed in accordance with Malterud. Every quotation was read and sorted into codes. In the second phase of the analysis the material was organized. Units, the quotations, with the same code were divided into defined main categories and categories. When appropriate the categories were divided into subcategories [17] The quotations could be placed in more than one category. However, each statement was only placed in one subcategory. During the whole process the findings were continually discussed in the research group in order to reach agreement. To validate the results, a sample of 50 quotations was randomly selected and re-analysed from the beginning of the analysis process. After consensus had been reached some of the quotations were transferred to other subcategories and three quotations were deemed irrelevant and removed. Those carrying out the analysis did not know the gestational week.

## Results

Four main categories and six subcategories were identified: “Frequency” (decreased frequency, absence of kicks and movement), “Intensity” (weaker fetal movements, indistinct fetal movements), “Character” (changed pattern of movements, slower movements) and “Duration”. The number in each category and subcategory as well as an presentation of the figures for women seeking health care in gestational week 28–32, gestational week 33–36 and during gestational week 37+, are shown in Table 2.

**Table 2:** Results

Women descriptions of fetal movement*	*N* = 876	Gestational week 28–32	Gestational week 33–36	Gestational week 37+
	n (%)	*n* = 190 (22)	*n* = 263 (30)	*n* = 423 (48)
I. Frequency	746 (85)	161 (85)	243 (92)	342 (81)
Decreased frequency of fetal movement	609 (69)	130 (68)	197 (75)	282 (67)
Absence of fetal movement	137 (16)	31 (16)	46 (17)	60 (14)
II. Intensity	343 (39)	85 (45)	111 (42)	147 (35)
Weaker fetal movements	277 (32)	65 (34)	90 (34)	122 (29)
Indistinct fetal movements	66 (8)	20 (10)	21 (8)	25 (6)
III. Character	252 (29)	46 (24)	70 (27)	136 (32)
Changed pattern of movements	141 (16)	36 (19)	34 (13)	71 (17)
Slower movements	111 (13)	10 (5)	36 (14)	65 (15)
IV. Duration	38 (4)	7 (4)	13 (5)	18 (4)

*The quotations could be placed in more than one category but each quotation was only placed in one subcategory

### Frequency

The most commonly experienced deviation of fetal movements concerned frequency, which was described in 746 (85 %) of the questionnaires. This category was divided into two subcategories; “Decreased frequency” and “Absence of kicks and movement”.

#### Decreased frequency of fetal movement

This subcategory comprises 609 (69 %) statements. These statements referred to movements becoming less frequent and indicating to the women a generally decreased liveliness in the fetus. The movements were described with words like, “a few”, “seldom”, “less frequent”, “not as many” and “decreased activity”.

*“Less frequent during the day”*

*“From being very active and kicking a lot to very few movements during some days”*

#### Absence of kicks and movement

Among the answers about the frequency of fetal movements, 137 (16 %) statements were about not feeling any movement at all.

*“I haven't felt any kicking for about 12 hours”*

“*Have not felt any movement during the whole day”*

### Intensity

A total of 343 (39 %) responses were perceptions that the movements had altered in intensity. Two subcategories were formed: “Weaker movements*”* and “Indistinct movements”.

#### Weaker fetal movements

This subcategory comprised 277 (32 %) statements. Words frequently used were: “Weaker”, “Softer”, “Less sharp” and “With less power”.

“*From obvious, strong movements and nudging to feathery tickling a few times a day”*

“*… The movements of the baby felt weaker the few times I have felt my baby”*

#### Indistinct fetal movements

Sixty-six (8 %) statements fell into this subcategory. Some women were uncertain as to whether they felt anything at all but thought they could imagine movements.

“*…The only thing I felt was non-specific movements deep inside my tummy…”*

“*Have previously felt apparent kicks which can be both felt and seen distinctly. Since yesterday evening only small occasionally twisting movements”*

### Character

This category comprised 252 (29 %) statements describing experiences of the fetal movements changing in character. The category revealed two subcategories: “Changed pattern of movements” and “Slower movements”.

#### Changed pattern of movements

This subcategory comprised 141 (16 %) statements. The women described the fetal movements as having changed in pattern and decreased in activity.

“*Not the same pattern of movements as before and not active at the same time”*

“*The baby has not moved at the times that she had moved earlier, following the pattern that she had previously. This has been going on for about 2 days. When she has moved, the movements felt weaker the past two days compared to before.”*

#### Slower movements

This subcategory included 111 (13 %) statements. When talking about the movements women used words such as: “sluggish”, “indolent”, “slow and sweeping”.

“*Calmer more tired movements as if it were tired…”*

“*Slow and smoother movements”*

### Duration

Thirty-eight (4 %) were included in this category. Women reported that the periods of movement had become shorter and had been reduced from several kicks in a row to occasional ones. However, the frequency of how often the baby had moved had not decreased.

“*… the periods when it has moved have been shorter”*

*“No more lively periods.”*

### Differences according to gestational age

Women in gestational weeks 33–36 experienced changes more often than women at term regarding the category Frequency (92 % vs. 81 %), the subcategory Decreased frequency (75 % vs. 67 %), and the category Intensity (42 % vs. 35 %). Compared to women at term, those in gestational weeks 28–32 expressed changes to a lesser extent within the category Character and the subcategory Slower movements (5 % vs. 15 %) (Table 2).

Four percent, 32/876, of the total number of women in this study only stated a change in the character of the movements, not included in any other category. The distribution regarding length of pregnancy was; gestational week 28–32, 1/190 (0.5 %), 33–36, 1/263 (0.4 %) and gestational weeks 37+, 30/423 (7 %). There were no statistically significant differences in the other categories (Not in table).

In addition to the responses categorised in accordance with the question, the women also mentioned how they had tried to stimulate the fetus to move and that they had difficulty in distinguishing fetal movements from contractions. Further, they described worry due to incidents related to changed pattern of fetal movements.

### Stimulation due to less movement

We identified 146 (17 %) statements about trying to provoke movement by triggering the fetus. Most of the women reported that they did this when not having felt movements for a while. When they did not succeed they consulted health care. The methods used to trigger movements were to pull, nudge or push on the tummy, stimulate with light or noise, take a shower or bath or to drink cold, sweet drinks. Others said that they had various positions they used to feel the baby more distinctly. Some women described not feeling movements without stimulating the baby.

*“No pushes” back when I am pulling on the tummy, no reaction when drinking a glass of lemonade. Otherwise he has been quite active and you have been able to see my tummy moving”*

“*Even if I touch my tummy, eat, drink, there is not much difference. He is moving considerably less”*

### Difficult to distinguish fetal movements from contractions

The women stated that the fetal movements ceased or changed in relation to contractions or that it was difficult to distinguish movements from contractions. Some women also described that the movements decreased in relation to contractions, pain in the tummy or the back. We identified 40 statements (5 %) concerning difficulties in distinguishing fetal movements from contractions.

“*Not felt any movements since the contractions became more intensive”*

“*It has been more difficult to perceive movements. Difficult to distinguish movements from contractions… previously the movements have been very distinct”*

### Worry due to incidents related to changed pattern of fetal movements

We identified 25 (3 %) statements about external factors, such as the woman was ill and perceived less fetal movement. Six women stated that they consulted health care due to pain in relation to changed patterns of fetal movement. Two statements referred to the woman having taken a fall and wanting to be reassured that the fetus had not been damaged. Other reasons related to increased worry were: post maturity, following an expelled mucus plug, an external cephalic version attempt, rupture of the membranes and previous stillbirth in the same gestational week.

*“Used to move a lot during both day and night. Have been ill with fever for three days and then there have been movements 4–5 times every twenty-four hours”*

*“Not as often as before but I still feel him daily. We’re extremely worried as we lost our first child in gestational week 33 in utero so it may be imagination”*

## Discussion

We are not aware of any studies that have categorized how women describe the changes they have perceived concerning fetal movements when they seek health care due to worry about their unborn baby.

Women who consulted health care due to decrease fetal movements described changes in frequency, intensity, character and duration of the movements. However, all women in this study were reassured after an examination of their unborn baby. In Norway, as many as 51 % of women reported that they were concerned about decreased fetal movements once or more in pregnancy [14]. In different populations, between four and 15 % consulted health care facilities because of decreased fetal movements in the third trimester [1]. There are several factors which may impair the ability to recognize fetal movements [8]. However, we have no data concerning amniotic fluid volume, fetal position, placenta position, smoking, overweight and nulliparity among the women participating in this study. These factors may explain some of the women’s perceptions of decreased fetal movements. Also, the plateau in gestational week 32 [3] may be perceived as a decrease. In a study by Sheikh and colleagues (2014), 729 women counted and registered fetal movements for one hour three times per day. Eight percent of the pregnant women in the third trimester, who in the end gave birth to a healthy child, experienced reduced fetal movements. Further, the researchers found that among women who consulted health care for reduced fetal movements but later gave birth to a healthy child, more of them were working than those who did not perceive reduced fetal movements [18]. We do not have data as to work status among the women participating in our study.

Placental dysfunction is one main reason for decreased fetal movements in late pregnancy [19]. It is thus important for the pregnant women to recognize the pattern of movement. A change may be a sign of asphyxia due to the redistribution of the circulation which gives priority to the brain over peripheral parts [20]. All fetuses in the present study were examined and no symptoms of asphyxia or placental dysfunction were identified at the time when the woman consulted health care. The women’s worry about their unborn baby’s health due to decreased fetal movements in this study did not result in a diagnosis or actions to induce the delivery.

Our results indicate that some women at term seek health care due only to a change in the character of the fetal movements. Although these women were asked to describe how their baby had moved less or differently, they did not mention a decrease in frequency in the fetal movements or a change in intensity. Slow, as in slow motion, stretching and turning, are descriptions of the character of fetal movements used by women in full term pregnancy, pregnancies that resulted in a healthy child [7]. The women in our study who consulted health care merely due to a change in the character of the movements and not because of altered frequency and intensity might not have been aware of normal changes in the fetal movement patterns in late pregnancy. The changes they reported as different can be physiological due to limited space in the uterus at term [3]. There is no routine in Swedish antenatal health care for giving information about fetal movements but women are recommended to consult health care if they experience decreased fetal movements [21]. However, pregnant women ask for information about fetal movements in general and for information about the number and type of fetal movements they can expect, as well as how the movements are supposed to change over time in pregnancy [22].

There were no stillbirths among the women in this study. Thus, we can only speculate that it is possible that women who consult health care due to decreased or changed patterns of fetal movement may be aware of the importance of detecting fetuses at risk as early as possible. Detection of decreased fetal movements can improve the outcome and reduce delay in consulting health care [23, 24]. Further, the fetuses in this study who could be at risk were examined and risk factors such as placental abruptions, growth retardation or malformations [25] may have been detected. The primary reason for consulting health care due to decreased fetal movements is worry about the health of the baby [14]. None of the women in our study consulted health care without cause, but their worry was obviously unfounded from a medical perspective in the short term.

### Strengths and limitations

Women in this study had a normal CTG before they completed the questionnaire. However, aside from no stillbirths among the participating women, we have no data regarding the health status of the baby after birth. This is a major limitation of the study. There is also only sparse information about the women’s’ sociodemographic background.

One strength of the study is the large number of participants. Another strength is that all delivery wards in Stockholm participated in the study. However, all women came from the capital city in Sweden where women in generally are older and well educated compared with women outside the capital. Further, the number of those who declined to participate and their reasons for doing so are not known.

The wording of the request, “Try to describe how your baby has moved less or had changes in movement” might have influenced the responders to use the words “decreased” and “differently” in their descriptions of their experiences. The results may have yielded even more if the initial request had been broader or more open, for example, “Try to describe how your baby has moved”. However, the context in which the women completed the questionnaire was one of already perceived decreased fetal movements.

### Clinical implications

Increased knowledge about the normal changes in the fetal movement patterns in late pregnancy can be one way to lessen the number of visits to obstetric clinics from women over concerns that turn out to be unnecessary from a medical perspective. The challenge from a clinical perspective is to inform and advise pregnant women about fetal movements with the goal of diminishing the length of pre-hospital delay if the fetus is at risk and at the same time reduce worry leading to unnecessary consultation. Reducing the pre-hospital delay when the intrauterine environment is a threat to the unborn baby’s life will provide a window of opportunity to save a greater number of children from death or compromised health. Further, fewer visits to obstetric clinics, over concern that turns out to be unnecessary from a medical perspective, will have health economic benefits. Before giving definitive advice that can reduce unnecessary controls at the end of the pregnancy, distinct differences must be identified regarding how women who lost their child intrauterine or have given birth to a hypoxic or anaemic child, report the changes in character of movements as only symptoms when they seek care for decreased fetal movements. Future studies are needed.

## Conclusions

Women reported changes in fetal movement concerning frequency, intensity, character and duration; they described decreased, absence, weaker, slower and changed pattern of the movements.

## Additional file

Additional file 1: A study on fetal movements, the complete questionnaire. (DOCX 33 kb)
