# Supplementary material for: Fetal movement in late pregnancy – a content analysis of women’s experiences of how their unborn baby moved less or differently
Source: BMC Pregnancy Childbirth. 2016 Jun 1;16:127. doi: 10.1186/s12884-016-0922-z (PMC4888620; doi:10.1186/s12884-016-0922-z)
Supplement: Additional file 1: — A study on fetal movements, the complete questionnaire. (DOCX 33 kb) [file 12884_2016_922_MOESM1_ESM.docx]

**Fetal movement after gestational week 28**

**1.** Are you seeking health care because you have felt that your baby has reduced movement or a change in movement?

🞐 Yes 🞐 No, I am seeking health care for ………………………….

**2.** What is the expected date of birth for your baby? (year-month-day)?..............

**3.** How many times previously (during your present pregnancy) have you sought health care because of reduced movement or changes in movement?

🞐 None

🞐 1 time

🞐 2 times

🞐 3 times

🞐 4 or more times

**4.** Try to describe **how** your baby has moved less or had changes in movement:

…………………………………………………………………………………………………………………………………………….…….

………………………………………………………………………………………………………………………………………….……….

…………………………………………………………………………………………………………………………………………….…….

…………………………………………………………………………………………………………………………………..................

……………………………………………………………………………………………………………………………………….………….

…………………………………………………………………………………………………………………………………….…………….

…………………………………………………………………………………………………………………………………………………..

…………………………………………………………………………………………………………………………………………………..

**5.** Who **recommended** you to come to the clinic?

Tick one of the boxes below for the alternative that best applies to you:

🞐 The midwife at the antenatal care center

🞐 The midwife at the labour ward

🞐 My partner

🞐 Another individual/other people (who?)……………………………………………….

🞐 **No one**, I have come on my **own initiative**

**6.** Why, specifically, do you come to the clinic **today**?

…………………………………………………………………………………………………………………………………………………..

…………………………………………………………………………………………………………………………………………………..

…………………………………………………………………………………………………………………………………………………..

**7.** Are there any reasons why you did not come to the clinic earlier?

…………………………………………………………………………………………………………………………………………………..

…………………………………………………………………………………………………………………………………………………..

…………………………………………………………………………………………………………………………………………………..

**8.** How **long** have you felt a reduction or change in fetal movements?

🞐 Less than 3 hours 🞐 About 2 days

🞐 About 6 hours 🞐 About 3 days

🞐 About 12 hours 🞐 About 4 days to 1 week

🞐 About 1 day 🞐 🞐 More than 1 week

**9**. How do you feel the movements **now**, **compared** to how you felt the

movements over the **past two weeks**?

|  | Do **not agree** at all | Agree in part | Agree **completely** |
| --- | --- | --- | --- |
| I think that the **number** of  movements has **decreased** |  |  |  |
| I think that **the number** of  movements has **increased** |  |  |  |
| I think that **the number** of  movements is about the **same** |  |  |  |
| I think that **the strength** of the movements **has decreased** |  |  |  |
| I think that **the strength** of the movements **has increased** |  |  |  |
| I think that **the strength o**f the movements is about the **same** |  |  |  |

**10.** **When** did you last feel your baby moving?

(Tick one of the boxes below for the alternative that best applies to you)

🞐 About 1 hour ago

🞐 About 2 hours ago

🞐 About 3 to 4 hours ago

🞐 About 5 to 6 hours ago

🞐 About 12 hours ago

🞐 About 18 hours ago

🞐 About 1 days ago

🞐 About 2 days ago

🞐 About 3 to 7 days ago

🞐 More than 1 week ago

**11**. What **type of fetal movements** have you experienced over the **past two days**.

|  | Do not **agree** at all | Agree in part | Agree **completely** |
| --- | --- | --- | --- |
| **Powerful movements:**  The movements felt strong and powerful |  |  |  |
| **Stretching movements:**  The movements felt as if the baby braced itself and then tried to stretch out |  |  |  |
| **Slow movements:**  The movements felt determined and slow |  |  |  |
| **Side-to-side movements:**  It felt as though the baby was turning from side to side |  |  |  |
| **Big movements:**  The movements felt really big as if the baby’s entire body was moving |  |  |  |
| **Light movements:**  The movements felt weak |  |  |  |
| **Jerky movements:**  The movements felt like twitching |  |  |  |
| **Hiccups:**  It felt as though the baby was hiccupping |  |  |  |
| **One episode of very strong and rapid movements:**  It felt as the baby was extremely active for a short time and then become motionless |  |  |  |
| I have **not felt any movements** from my baby during **the past two days** |  |  |  |
| Describe what you have felt during **the past two days:** | | | |

**12.** Have you experienced **contractions** during **the past month?**

|  | **Never** | About  **every week** | About  **every day** |
| --- | --- | --- | --- |
| Powerful contractions |  |  |  |
| Light contractions |  |  |  |
| Regular contractions |  |  |  |
| Irregular contractions |  |  |  |

**13**. **How** have you observed your baby’s movements during **the past month**?

|  | **Never** | About **every**  **week** | About **every day** |
| --- | --- | --- | --- |
| I have tried to **concentrate** on the baby’s movements for a moment |  |  |  |
| I have **counted** the number of movements  during a specific time (for example 10 – 15 minutes) |  |  |  |
| I have checked the length of time it takes for my baby to move ten times |  |  |  |

**14.** How have you explicitly felt your baby during **the past month**?

Tick the box for the statement that best applies to you.

|  | Do not agree  at all | Agree in part | Agree **completely** |
| --- | --- | --- | --- |
| I distinctly felt my baby |  |  |  |
| I had difficulty telling when my  baby was awake |  |  |  |
| It was easy for me to know when my baby was awake |  |  |  |
| My baby moved lightly throughout  the month |  |  |  |

**15.** Which side do you **usually** lie on in bed when you are going to sleep?

🞐 On my back

🞐 On my right side

🞐 On my left side

**16.** Which side did you lie on when you were going to sleep **last night**?

🞐 On my back

🞐 On my right side

🞐 On my left side

**17.** What is your highest level of education?: 🞐 Primary school

🞐 High school or equivalent

🞐 University or college 1-3 years

🞐 University or college > 3 years

**18.** In what country were you born?

🞐 Sverige

🞐 Scandinavia (not Sweden)

🞐 Europe (not Scandinavia), country:………………………………………………………….

🞐 Asia, country:………………………………………………………………………………….……………

🞐 Africa, country:……………………………………………………………………………………………

🞐 South America, country:……………………………………………………………………………

🞐 North America/Canada

🞐 Australia/New Zealand

**19.** Your personal identity number (year-month-day- and the last 4 digits)

(We require this information in order to provide a follow up after your baby is born)

…………………………………………………………………………………………………………………………………………………..

**20**. The date on which you completed the questionnaire (year-month day)……………

**21.** Write feely here, your thoughts and comments, to the health care professionals working with pregnant women who feel a reduction or change in their baby´s movements.

…………………………………………………………………………………………………………………………………………………

…………………………………………………………………………………………………………………………………………………

…………………………………………………………………………………………………………………………………………………

…………………………………………………………………………………………………………………………………………………..

…………………………………………………………………………………………………………………………………………………..

**22.** Have you any advice to give to other pregnant women who may feel a reduction or change in their baby´s movements?

…………………………………………………………………………………………………………………………………………………

…………………………………………………………………………………………………………………………………………………

…………………………………………………………………………………………………………………………………………………

…………………………………………………………………………………………………………………………………………………..

…………………………………………………………………………………………………………………………………………………..

**Thank you for your participation!**

Please place the completed questionnaire in the provided envelope and seal it, give the envelope to the midwife or physician you meet at the hospital.

If you have any comments, questions or suggestions, you are very welcome to contact the research group. The contact information is available in the introduction letter you received.
